# Being Eaten Alive: How Energy-Deprived Cells Are Disposed of, Mediated by C-Reactive Protein—Including a Treatment Option

**DOI:** 10.3390/biomedicines11082279

**Published:** 2023-08-16

**Authors:** Ahmed Sheriff, Rudolf Kunze, Patrizia Brunner, Birgit Vogt

**Affiliations:** 1 Department of Gastroenterology, Infectiology, Rheumatology, Charité University Medicine Berlin, 10117 Berlin, Germany; 2 Pentracor GmbH, 16761 Hennigsdorf, Germanybrunner@pentracor.de (P.B.); vogt@pentracor.de (B.V.)

**Keywords:** AMI, apoptosis, complement, COVID-19, apheresis, hypoxia, infarct size, phagocytosis

## Abstract

In medicine, C-reactive protein (CRP) has become established primarily as a biomarker, predicting patient prognosis in many indications. Recently, however, there has been mounting evidence that it causes inflammatory injury. As early as 1999, CRP was shown to induce cell death after acute myocardial infarction (AMI) in rats and this was found to be dependent on complement. The pathological effect of CRP was subsequently confirmed in further animal species such as rabbit, mouse and pig. A conceptual gap was recently closed when it was demonstrated that ischemia in AMI or ischemia/hypoxia in the severe course of COVID-19 causes a drastic lack of energy in involved cells, resulting in an apoptotic presentation because these cells cannot repair/flip-flop altered lipids. The deprivation of energy leads to extensive expression on the cell membranes of the CRP ligand lysophosphatidylcholine. Upon attachment of CRP to this ligand, the classical complement pathway is triggered leading to the swift elimination of viable cells with the appearance of an apoptotic cell by phagocytes. They are being eaten alive. This, consequently, results in substantial fibrotic remodeling within the involved tissue. Inhibiting this pathomechanism via CRP-targeting therapy has been shown to be beneficial in different indications.

## 1. Introduction

The scientific knowledge about C-reactive protein (CRP) has evolved dramatically since its first description in 1930, where it was named for its obvious appearance reacting to the C-component in pneumococci-infected blood [1]. After being characterized as the first acute-phase protein and substantially forming the whole field of the acute phase response, it was then, for several decades, primarily known for its rather broad response to any kind of bacterial and viral infection or inflammation within the human body [2]. Synthesized in the liver upon inflammatory stimuli, it is released into the blood stream as a homopentamer [3,4]. Up to 1000-fold elevated levels can be detected following an acute phase stimulus, which led to its wide-spread use as an exceptionally robust, accurate and early biomarker of inflammation [5,6]. Eventually, it became clear that the actual biological function of CRP was to opsonize certain pathogens and apoptotic/necrotic cells and mark them for disposal; thereby, CRP functions as a substantial part of the innate immune system and represents part of the primary systemic response.

CRP initiates the phagocytosis of, e.g., bacteria and also of compromised host cells [6]. This is beneficial for removing pathogens and also for removing inflamed, injured tissue, which might be the ground for new infections, specifically within external wounds. In the setting of sterile tissue inflammation, which is defined by inflamed tissue without external pathogens, CRP’s action is perilous. Here, cells are impaired by, e.g., oxygen-deprivation (heart attack, stroke) [7,8]. The lung damage present in COVID-19 patients can be described similarly as it is primarily produced by excessive edema-induced inflammation [9,10]. The initial incident triggers high CRP levels, which then lead to the disposal of impaired cells.

In this setting, CRP-targeting therapies are hypothesized to be beneficial [11,12,13,14]. Selective immunoadsorption of CRP from blood plasma (“CRP apheresis”) is the only approved and routinely used specific CRP-targeting therapy in the clinic up to now [15]. It achieves a rapid and efficient reduction of the fulminant CRP burden [15,16].

From this perspective, we summarize and discuss the new findings concerning the rapidly emerging CRP-apheresis therapy and their clinical implications.

## 2. CRP in Pathogenesis

CRP is highly evolutionary conserved and present in virtually all species, where it forms immune complexes together with complement proteins. This happens already in Limulus, a species which is a minimum of 250 million years old [17,18]. Specifically, in species without an adaptive immune system, e.g., fish, CRP can be phylogenetically considered as an antibody of the innate immune system. This has prompted the suggestion that CRP represents a prehistoric precursor to antibodies in mammals, which evolved much later.

In humans, like antibodies, CRP triggers the complement system through the classical pathway and macrophages by attaching to Fc receptors, similarly to antibodies, and thereby labels pathogens for phagocytosis [19,20,21,22,23]. Hence, CRP efficiently recognizes and opsonizes bacteria as they infiltrate tissue [24,25]. The additional role of CRP as a trigger of tissue damage has long been neglected, even though there has been clear evidence for this in some animal species [13,26,27,28]. Both actions are based on the same molecular basis.

CRP binds with a high affinity to certain lipid moieties (phosphocholine groups) present in bacterial cell walls, but also in all human cell membranes [29,30]. The phosphocholine groups are not accessible to CRP’s binding site in healthy mammalian cells due to their conformation. In contrast, cells which are apoptotic, necrotic, in energy shortage or simply localized in an inflammatory environment (i.e., in a hypoxic or acidic environment) expose phosphocholine groups through conformational and biochemical changes in their membrane [31,32,33]. Briefly, partial hydrolysis of phosphatidylcholine (PC) results in the formation of lysophosphatidylcholine (LPC), a process catalyzed by secretory phospholipase A2 type IIa (sPLA2 IIa), which is also an acute-phase protein [32,34,35,36]. LPC allows for the binding of CRP, which irreversibly labels activated, dead, dying, damaged, ischemic or hypoxic cells. This is followed by activation of the classical complement pathway up to C4, which leads to phagocytosis of the CRP-labeled cells [37,38,39,40,41,42,43]. It has been shown that CRP specifically promotes the clearance of apoptotic cells, while protecting cells from necrotic cell death by inhibiting the assembly of the terminal complement attack complex [44].

**Figure 1:**
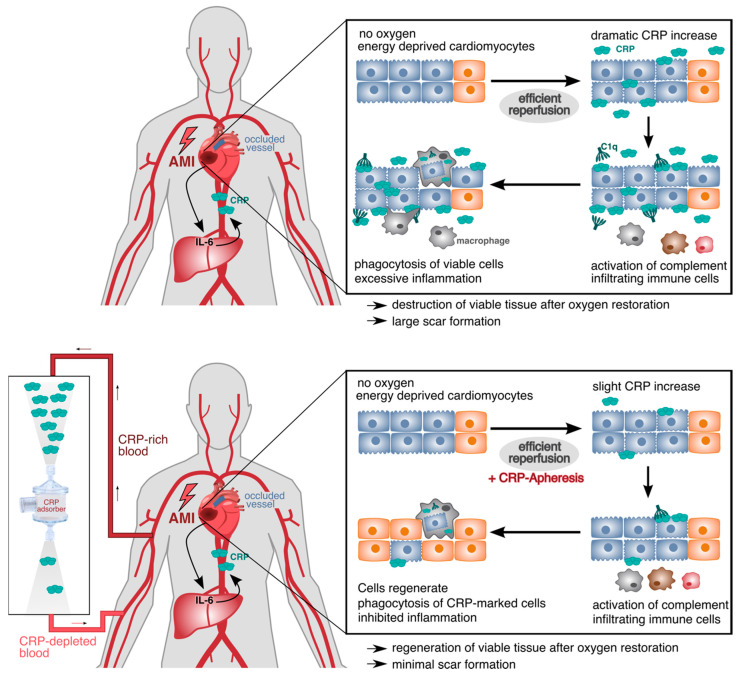
CRP-mediated damage in ischemic tissue after acute myocardial infarction (AMI). Energy-depleted cardiomyocytes after AMI temporarily change their energy metabolism and exhibit molecular changes in their membrane (blue cells). Upon efficient reperfusion, the oxygen supply is restored, and anaerobic cells can reverse their metabolism and the molecular membrane changes (orange cells). CRP increases dramatically in the first 24 h after AMI and binds to LPC within the modified membranes of impaired cardiomyocytes. CRP opsonizes the cells and triggers the activation of the classical complement pathway starting with C1q. This leads to the phagocytosis of still viable cells by infiltrating immune cells (**upper panel**). With CRP-apheresis, the dramatically rising levels of circulating CRP can be depleted, and cells acquire the time to regenerate their metabolism and membrane after reperfusion. They are not marked due to lack of CRP and can contribute to healthy myocardial tissue again (**lower panel**).

Connected with acute injury such as an acute myocardial infarction, this creates a vicious circle: the primary inflammation, induced by acute organ ischemia, causes a marked synthesis and secretion of IL-6 and, consecutively, CRP. In the ischemic tissue, there is no immediate tissue necrosis, but a switch of energy metabolism to anaerobic glycolysis, leading to a marked shortage of energy in the individual cardiomyocytes. After this, the cardiomyocytes go into a state of hibernation (myocardial hibernation) or stunning up to the time when metabolism returns to the aerobic mode, which resolves the energy shortage [45,46]. In principle, these cardiomyocytes survive only if they are not labeled by CRP and are consequently removed by phagocytes. High CRP concentrations finally reach the wound area and mark the still viable cells, which could potentially regenerate their membrane, following reestablishment of the oxygen flow and conversion to aerobic metabolism (i.e., 16-fold greater energy production per glucose molecule). Phagocytosis of these cells leads to further production of IL-6, consecutive CRP and further amplification of the immune response. Through these mechanisms, CRP contributes in a causal way to tissue damage and scar formation in the context of acute organ ischemia [8,47,48,49]. This disposal of vital anaerobic cells occurs via phagocytosis. These cells are eaten alive (Figure 1).

CRP has proven to be one of the most reliable markers of inflammatory processes and increases significantly with any type of inflammation. At the forefront of recent research are numerous studies on the role of CRP as a marker of systemic inflammation, in which it is moderately but chronically enhanced [50,51,52,53]. Few studies also infused CRP into human subjects in order to investigate whether CRP mediates these systemic inflammation actions. Controversially, CRP has been shown to activate inflammation and coagulation in some studies [54], while it had no effect on cytokine levels in others [55]. While the action of chronically enhanced levels of CRP in healthy subjects is yet to be determined, its pro-inflammatory effects in individuals with existing tissue damage are clear. Current aspects of CRP research focus on biological functions of the CRP pentamer and its dissociated form, the CRP monomers [56,57,58,59]. The conversion of pentameric CRP to monomeric CRP has been observed in specific inflammatory microenvironments [56,59]. Circulating pCRP is activated by binding to the altered plasma membrane of a cell, leading to a partially dissociated pentamer (pCRP*), which eventually dissociates into monomeric CRP (mCRP) [60,61]. These monomers are then supposedly highly pro-inflammatory, binding complement, neutrophils and activating platelets [61,62,63,64,65]. It is hypothesized that this can be also induced by decreasing pH and circulating immune cells, thereby augmenting pro-inflammatory actions of CRP even if ischemic/damaged cells are not abundant and hence leading to tissue damage of adjacent healthy tissue [66,67].

Although circulating CRP secreted by the liver is always pentameric and provides the sole source for mCRP, these recent findings add another layer to CRP’s action in tissue destruction and substantiate the detrimental role CRP plays in inflammation-mediated cell death.

## 3. CRP Causes the Disposal of Ischemic and Hypoxic Cells

### 3.1. Evidence from Myocardial Infarction

The therapeutic goal in patients with acute myocardial infarction (AMI) is to reopen the affected vessel as quickly as possible in order to prevent or reduce the extension of the infarction. Reperfusion is generally performed via primary PCI and/or fibrinolysis in accordance with European Society of Cardiology guidelines for AMI. The greater the remaining damage, the higher the risk of serious subsequent complications (e.g., heart failure, cardiac arrhythmias, second heart attack, death) and impairment of the patient’s quality of life [68,69]. In this context, the mortality rate and occurrence of secondary complications correlates directly with the extent of myocardial damage and scarring [70,71]. Illness-related death or damage mean an enormous financial burden for the healthcare system.

It has long been known that the initial damage to the myocardium is significantly exacerbated by the activation and subsequent action of the innate immune system, even after reperfusion [72,73,74,75,76,77]. Specifically, the rising CRP concentration after an acute myocardial infarction correlates significantly with the clinical outcome of the patient [48,78,79,80,81,82,83,84]. Likewise, the importance of the initial rise of CRP levels in the first ~48 h for prognosis is reported in several publications [79,84,85,86].

These clinical observations agree well with the aforementioned pathological effect of CRP to mark cells in the “risk area” of a myocardial infarction wound for later elimination. The “area at risk” of an infarct wound comprises cells that could in principle recuperate after revascularization and reperfusion, but which are prematurely disposed of by immune-mediated mechanisms, primarily via apoptotic mechanisms [10,87]. In view of these observations, it was suggested several years ago that elevated CRP levels in acute myocardial infarction should be specifically reduced. However, therapeutic approaches were used or suggested whose effect was clinically irrelevant or only set in with a delay [12,28,88].

The development of CRP immunoadsorption solved these problems, and its principle efficacy was first shown in a preclinical study in pigs, in which a significant reduction of the infarct area and a consecutive stabilization of the left ventricular ejection fraction (LVEF) could be achieved [13]. Moreover, after CRP apheresis, the scar morphology of the animals looked entirely different from that of the sham-treated control animals, an observation that supports the notion that CRP is actively participating in post-infarction tissue destruction and scarring [13].

CRP apheresis was then used in a non-randomized clinical trial after acute myocardial infarction (CAMI-1 trial) [84]. It was first established in this patient cohort that the magnitude of the increase in CRP concentration in the 32 h after ST-elevation myocardial infarction (STEMI) correlated significantly with infarct size in control patients. Thus, there was a distinct dose-response relationship between the CRP increase and the myocardial infarction damage. Compared with control patients, patients with a comparable initial CRP elevation (before CRP apheresis) who received CRP apheresis had a lower infarct size and better LVEF and ventricular wall motion. This effect was most pronounced in patients with high CRP. Remarkably, several patients who had received CRP apheresis had no infarct scars whatsoever and a concordantly normal LVEF. This leads to the assumption that the infarct event itself is not yet the cause of the damage. After 12 months, three serious adverse cardiac events occurred in the control group, with no events in the apheresis group. Overall, the apheresis treatments were well tolerated by the myocardial infarction patients [84].

It was also shown that patients with high CRP synthesis rates after AMI benefit most from CRP-targeting therapy and that the reduction of myocardial damage, assessed by infarct size and LVEF in cardiac magnetic resonance imaging (CMR), was sustained after scar formation is considered complete [89].

These results are very promising and drive CRP-apheresis to the forefront of novel AMI therapies improving regeneration after reperfusion.

### 3.2. Evidence from Severe COVID-19

Infection with SARS-CoV-2 can lead to COVID-19. A small percentage of infected individuals develop pulmonary fibrosis and cardiac (among other) complications [90]. A key therapeutic strategy is focused on the management of acute respiratory failure, the main cause of death from COVID-19, succeeded by cardiac and septic complications. This is mainly targeted by corticosteroids, IL-6 receptor blockers and Baricitinib because COVID-19 is a highly immunologically driven disease with severe systemic hyperinflammation [91].

The serious course of the disease is accompanied by a dramatic elevation of CRP, combined with a cytokine storm, which may then develop into pulmonary fibrosis [92,93,94]. Intra-alveolar edema and hemorrhage are a frequent phenomenon observed in the lungs of COVID-19 patients, leading to ischemic alveolar tissue [9,95,96]. Based on the molecular mechanism explained above, CRP causes tissue damage by binding to ischemic cells and is hence causally participating in the magnification of irreversible lung tissue damage in COVID-19 [97]. The levels of both IL-6 and CRP rise substantially during the fulminant course of COVID-19 [95], with sharply rising CRP levels often preceding the patient’s respiratory collapse [93]. CRP levels further significantly correlate with the extent of lung involvement in chest CT findings of COVID-19 patients [98]. Correspondingly, CRP and complement deposits, most notably C1Q, were found in the lungs of deceased COVID-19 patients [99,100]. CRP binds C1Q after binding to LPC on ischemic cells [101]. The scientific consensus 3 years after the beginning of the SARS-CoV-2 pandemic is that this increased CRP plasma concentration correlates inversely with the patient outcome [93,94,102,103,104,105,106,107]. The odds ratio (strength of the correlation) for mortality has been shown to increase with the level of CRP, rising sharply to more than 23 at a CRP level of >250 mg/L [106]. These findings underpin the hypothesis that a significant elevation of CRP in the blood of COVID-19 patients is an indicator of impending lung deterioration and thus disease progression.

Very impressively, Esposito et al. [108] demonstrated the benefits of eliminating CRP in severe COVID-19. The therapy was administered as part of the C-Reactive Protein Apheresis in COVID (CACOV) registry to enable a scientific assessment in this setting (DRKS00024376). It is a compelling report on a hardly known therapeutic option for severe COVID-19, with remarkable results shown in lung CT and X-ray images. Besides the fact that the mortality rate was incredibly low in a group of patients in which a rate of more than 40% was anticipated, the damage to the lungs, which normally progresses, was reversed. Likewise, the expected 50% mortality rate within 12 months following hospitalization was not observed among this cohort [108,109]. A further series of cases out of the same registry (CACOV) reporting similar findings has been published by a different clinic [110,111]. These reports affirm that cells suffering from oxygen deficiency are being cleared via CRP despite still being vital, and that this tissue damage indeed does not take place if a substantial amount of CRP is cleared from the blood plasma.

## 4. CRP-Targeting Strategies

Experimental CRP-targeting strategies have been suggested and investigated in numerous indications, including cardiovascular incidents (AMI, ischemic stroke), hyperinflammatory settings (COVID-19, pancreatitis) and autoimmune diseases (rheumathoid arthritis [112], Crohn’s disease [10]). They do not only represent a potential benefit for the patient but also serve as proof of concept of the pathophysiology of CRP’s action in these indications. The first anti-CRP molecule, bisPC, composed of two phosphocholines with a hexane linker (1,6-bis(PC)-hexane), has been investigated in a rat AMI model [12]. Here, bisPC was shown to abrogate the detrimental effects of infusing human CRP when applied 2 days before inducing an infarct. This unfeasible acute care protocol, as well as the problem of administrating low-molecular-weight inhibitors in humans, has stopped this molecule from being a therapeutic option. While the suggestion to strategically target CRP therapeutically has been around since then, no other treatment options besides CRP-apheresis are approved for use in humans and available in the clinical setting.

Since the first AMI patient was treated with CRP-apheresis 8 years ago, it has been successfully and safely used in patients suffering from AMI, acute necrotizing pancreatitis, Crohn’s disease, COVID-19, stroke and after bypass surgery. Up to April 2023, 669 apheresis treatments have been documented for 229 patients within five clinical studies and two clinical registries in Germany. Over all apheresis treatments, only 26 application-related adverse events were reported (3.9%), of which most represented CTC grade 1 symptoms related to the venous access (thrombosis, edema, pain) or the general process of plasma separation, anti-coagulation and re-infusion (blood pressure drop, dizziness, headache). Only five apheresis treatments had to be aborted. No serious adverse events related to the apheresis procedure have been reported so far. No immune-compromising effects have been observed in any of these patients.

Recently, based on the original bisPC, a new inhibitor (C10M) which mimics PC and interacts with the PC-binding site of pCRP was used and studied in rats. Here, the authors argue that the maintenance of A- and B-surface binding of CRP to other proteins is advantageous and speaks to the specificity of their compound in only inhibiting PC binding and not CRP-dependent immune responses in general [61]. However, it remains to be shown which CRP functions can still be executed while bound to C10M and whether those are not also pro-inflammatory and should not, therefore, also be inhibited.

The main advantage of a therapy such as CRP-apheresis is not introducing a pharmaceutical compound, which can always harbor potential side effects or complications. With a PC-mimic, one has to consider the administration of a highly polar, small molecular compound with poor bioavailability and a rapid half-life in high molecular access [12,15,61].

With all CRP-targeting strategies, one has to discuss the potential immune compromising effects they could elicit. Based on all publications and clinical experiences so far, they are neglectable. Whether CRP is depleted by apheresis or inhibited by a small molecule, its circulating and acting concentration never reaches 0 mg/L. As the classical acute-phase protein, CRP synthesis reacts to its triggers within hours and can increase manifold rapidly. In healthy bodies, circulating CRP levels are constantly <5 mg/L, and CRP has to be produced upon a novel trigger. Additionally, for CRP-apheresis, the treatment indications realized so far are acute and life-threatening settings such as AMI, stroke or severe COVID-19. Here, patients are hospitalized under constant care and treated for a short period of time. CRP synthesis under CRP-apheresis is not impaired, and the liver can react normally to potential infections. Further, in the hospital, such an infection would not go unnoticed and would be treated immediately with antibiotic therapy.

Recent studies on specific cancer types (urothelial carcinoma and non-small cell lung cancer) showed that CRP kinetics could predict the response to immunotherapy [113,114,115,116]. CRP flares after immunotherapy correlated significantly with prolonged survival and reduced tumor size. The eligibility of patients undergoing cancer immunotherapy for CRP apheresis has to be investigated.

Based on the existing data, we can assume that targeting CRP is beneficial in the described indications, and especially for sterile inflammation, as no advantageous effect of CRP on tissue regeneration has been described so far.

## 5. Summary and Outlook

Research activities in the field of C-reactive protein have undergone three fundamental changes. First, CRP was discovered as a general biomarker for inflammation and infection and established in clinical practice. Then, CRP, which is often present in chronically minimally elevated concentrations, was elaborated as a stable and prognostic factor for cardiovascular and cerebral diseases in healthy individuals. In the third step, CRP was identified as a mediator of tissue destruction in humans. As a primordial protein of the innate immune system, CRP initiates the disposal of cells and responds to almost any change in tissue homeostasis. From the perspective of an optimally designed energy balance of the body, it seems difficult to understand in the first place why the body generates large amounts of CRP in the liver during significant organ ischemia. Evolution certainly did not intend it to be merely a useful biomarker for contemporary medicine. More plausible is the hypothesis that after significant organ ischemia, the affected tissue has to be eliminated to avoid secondary infection. This is highly beneficial for external wounds but is harmful for internal aseptic wounds such as heart or brain infarcts or hypoxic lungs. Stunningly, energy-depleted vital cells are disposed of alive by phagocytes because of their labeling with CRP and its complement.

The recognition of CRP as a mediator or active inflammatory protein offers the promising possibility of removing CRP from the body quickly and efficiently in the case of excessive inflammatory reactions, thereby significantly counteracting tissue destruction. Current and future clinical trials and registries will provide further information on whether this new therapeutic approach will bring lasting benefits to patients in a number of indications.

## Data Availability

Data sharing not applicable.
